# Overall childbirth experience: what does it mean? A comparison between an overall childbirth experience rating and the Childbirth Experience Questionnaire 2

**DOI:** 10.1186/s12884-023-05498-5

**Published:** 2023-03-14

**Authors:** Frida Viirman, Susanne Hesselman, Inger Sundström Poromaa, Agneta Skoog Svanberg, Anna Wikman

**Affiliations:** 1 Department of Women’s and Children’s Health, Uppsala University, Akademiska Sjukhuset, 75185 Uppsala, Sweden; 2 Centre for Clinical Research Dalarna, Nissers Väg 3, 79182 Falun, Sweden

**Keywords:** Childbirth experience, Birth satisfaction, Labour, Psychological birth trauma, CEQ2, Questionnaires, VAS, Visual Analogue Scale, Single-item

## Abstract

**Background:**

In clinical settings and research studies, childbirth experience is often measured using a single-item question about overall experience. Little is known about what women include in this rating, which complicates the design of adequate follow-up, as well as the interpretation of research findings based on ratings of overall childbirth experience. The aim of this study was to examine which known dimensions of childbirth experience women include in the rating on a single-item measure.

**Methods:**

Ratings of overall childbirth experience on a 10-point numeric rating scale (NRS) from 2953 women with spontaneous or induced onset of labour at two Swedish hospitals were evaluated against the validated Childbirth Experience Questionnaire 2 (CEQ2), completed on one of the first days postpartum. The CEQ2 measures four childbirth experience domains: *own capacity, perceived safety, professional support* and *participation.* Internal consistency for CEQ2 was evaluated by calculating Cronbach’s alpha. NRS ratings were explored in relation to CEQ2 using empirical cumulative distribution function graphs, where childbirth experience was defined as negative (NRS ratings 1–4), mixed (NRS ratings 5–6) or positive (NRS ratings 7–10). A multiple linear regression analysis, presented as beta coefficients (*B*) and 95% confidence intervals (CI), was also performed to explore the relationship between the four domains of the CEQ2 and overall childbirth experience.

**Results:**

The prevalence of negative childbirth experience was 6.3%. All CEQ2-subscales reached high or acceptable reliability (Cronbach’s alpha = 0.78; 0.81; 0.69 and 0.66, respectively). Regardless of overall childbirth experience, the majority of respondents scored high on the CEQ2 subscale representing *professional support.* Overall childbirth experience was mainly explained by *perceived safety* (*B* = 1.60, CI 1.48–1.73), followed by *own capacity* (*B* = 0.65, CI 0.53–0.77) and *participation* (*B* = 0.43, CI 0.29–0.56).

**Conclusions:**

In conclusion, overall childbirth experience rated by a single-item measurement appears to mainly capture experiences of *perceived safety,* and to a lesser extent *own capacity* and *participation*, but appears not to reflect *professional support*. CEQ2 shows good psychometric properties for use shortly after childbirth, and among women with induced onset of labour, which increases the usability of the instrument.

**Supplementary Information:**

The online version contains supplementary material available at 10.1186/s12884-023-05498-5.

## Background

Childbirth experience is a woman’s subjective perception of the events and feelings connected with the birthing process. A positive childbirth experience can empower the mother, and has the potential to strengthen her self-esteem and self-confidence also in the long-term [1]. Negative childbirth experience, on the other hand, has a prevalence of almost 10% in high-income countries [2–4] and is linked to postpartum depression and post-traumatic stress disorder, both serious conditions, which can affect the mother and her new family for a substantial amount of time [5–7]. Moreover, negative childbirth experience is strongly associated with fear of childbirth. Fear of childbirth, which in itself is a psychological burden, often leads to requests for caesarean section (CS) in future pregnancies, but may also cause delay, or avoidance, of subsequent pregnancies [8].

In quantitative research, childbirth experience is often measured by a single-item question about overall childbirth experience, or about global perception of childbirth, rated on a 10-point numeric rating scale (NRS), a visual analogue scale (VAS) or a 5-point Likert scale [9–12]. Depending on study design, the definition of negative overall childbirth experience varies from ratings equivalent to ≤ 3 to ratings ≤ 5 on a 10-point scale, since no established cut-off exists [2, 13–15]. In Sweden and Finland, mothers rate overall childbirth experience on NRS or VAS as part of clinical routine at the birth clinics before discharge from the hospital. In Sweden, the childbirth experience score is registered in the hospital medical records and later transferred to a national quality register (the Swedish Pregnancy Register). Further, women who score low on childbirth experience are usually offered support such as postpartum counselling, according to local guidelines [3, 16].

VAS is a reliable and valid method for estimating pain and other feelings [17], but the scale is not designed for measuring childbirth experience. When validated against the more comprehensive and well-established instrument Wijma Delivery Experience Questionnaire B, designed to evaluate childbirth experience focusing on fear of birth, a modest correlation of 0.52 was observed [15, 18]. The practice of measuring childbirth experience with a single global rating has been questioned, since it is not known which aspects of the multifaceted childbirth experience women include in their overall rating, making interpretation of research findings and design of adequate follow-up after negative experiences challenging [19, 20].

A plethora of instruments for measuring childbirth experience have been developed over the years, several used only in single studies. In a systematic review, 36 instruments were assessed regarding quality of psychometric properties, to provide an overview of instruments suitable for use in research. Of the seven instruments considered to be reliable and valid, four specifically evaluate aspects known to have significant impact on the childbirth experience, such as support, communication, personal relationships, safety, participation and respect [21]. One of them was the Swedish-developed Childbirth Experience Questionnaire 2 (CEQ2) [22, 23].

Since overall childbirth experience, measured by NRS or VAS, is commonly used in research and in clinical settings in several countries, there is a need for validation of single-item global measurements against an instrument covering relevant elements of childbirth experience. To the best of our knowledge, this has only been done in two previous studies. However, these were limited by small study populations, and used the previous version of the CEQ [24, 25]. Interventions to prevent effects of negative childbirth experience are currently difficult to develop based on findings from studies of overall childbirth experience, as we have little understanding of what the measurement actually captures. Therefore, the aim of this study was to examine which known dimensions of childbirth experience women include in the rating of overall childbirth experience on a single-item NRS measure.

## Methods

### Study design and setting

This was a cross-sectional study conducted at two birth clinics in Sweden, one at a university referral hospital with 4000 births annually, and one at a regional hospital with approximately 3000 births annually. At both birth clinics, according to clinical routine, women are asked to rate overall childbirth experience after giving birth, which around 90% of women with spontaneous or induced onset of labour usually do.

### Data source and procedure

Eligible for participation in the study were women, aged 18 years or older, who gave birth to a live infant, and rated overall childbirth experience at the participating hospitals during the study period. An additional inclusion criterion was the ability to understand spoken and written Swedish. Exclusion criteria were elective CS and unplanned CS before onset of labour, since women giving birth in this way did not have experiences of labour, which prevented them from responding to most CEQ2 items. Recruitment lasted September 2020–December 2021 at the university referral hospital, and September 2021–January 2022 at the regional hospital, where the study start was delayed due to COVID-19 pandemic restrictions.

Information on overall childbirth experience was collected according to clinical routine one of the first days postpartum at each hospital. At the university referral hospital, women rated overall childbirth experience on a paper-and-pencil questionnaire with the instruction “Please mark the number best corresponding to your overall childbirth experience”, followed by an NRS 1–10, with anchors “1 = very poor experience” and “10 = very good experience”. At the regional hospital, women were asked orally which number between one and ten best corresponded to their overall childbirth experience. The same anchors were used at both hospitals.

The CEQ2 was used to measure childbirth experience more extensively. The questionnaire encompasses 22 items, representing four domains of childbirth experience. *Own capacity* includes experiences of capability, pain and control, *perceived safety* encompasses sense of security, fear and memories from childbirth, *professional support* corresponds to the midwife’s presence, attitude and behaviour, and *participation* includes items about shared decision making during labour and childbirth [23]. Nineteen of the items are statements with Likert scale response alternatives (totally agree, mostly agree, mostly disagree and totally disagree). Three items are ratings on a VAS of level of perceived pain, control and sense of security during labour. All answers are transformed into numerical values 1–4 for each item, and mean values are calculated for total CEQ2 score and for the subscales, representing four domains of childbirth experience. All scales range from 1 to 4, with higher values indicating a better experience. In the present study, subscale scores were calculated only if the respondent had answered at least half of the items included in the subscale. This is in line with the instructions for computing CEQ2 subscale scores, and missing data on individual questions were hence allowed. No imputation of missing values was performed.

At the time of rating of overall childbirth experience at the hospitals, women also received the CEQ2, together with a letter informing them about the study and inviting them to participate, from midwives and assistant nurses at the postnatal ward. Participants were informed that by completing the questionnaire they consented to participating in the study. Participants completed CEQ2 as a paper-and-pencil questionnaire. Socio-demographic and obstetric background information was collected by seven study-specific questions. Additional information on sociodemography, health history and childbirth was retrieved from the Swedish Pregnancy Register [16] and from electronic medical records. Ethical approval for the study was obtained from The Swedish Ethical Review Authority, Dnr 2019–06534, amendment 2020–05397 and 2021–03580.

### Statistical analysis

Sample characteristics and prevalence of negative childbirth experience, defined as NRS ratings 1–4, in line with previous studies [2, 14], were calculated using descriptive statistics, and presented as means and standard deviations (SD) or numbers (n) and percentages (%). Internal consistency for CEQ2 in this setting was evaluated by calculating Cronbach’s alpha (α) for subscales and the total scale. Higher α-values indicate greater internal consistency. There is no established cut-off for when reliability is achieved, but values above 0.70 are usually considered satisfactory [26].

**Fig. 1:**
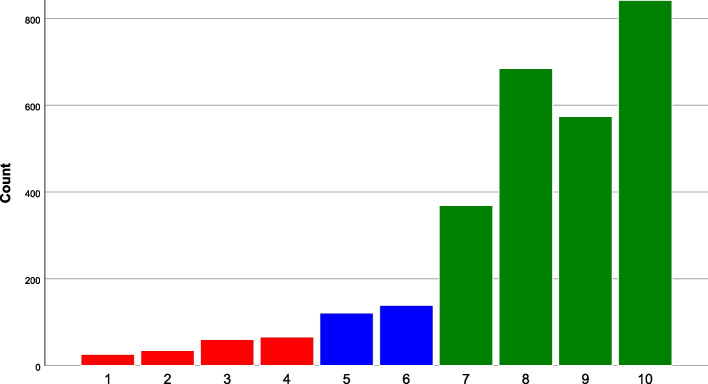
Distribution of overall childbirth experience (1–10) illustrating negative (red), mixed (blue) and positive (green) experience

To enable visual exploration of ratings of overall childbirth experience in relation to CEQ2 ratings, empirical cumulative distribution function (CDF) graphs were created using R Statistical Software version 4.2.1 (R Core Team, Vienna, Austria). Firstly, NRS ratings were categorised as negative, mixed, or positive. The categorisation was made after visual inspection of the distribution of NRS values, where three levels of overall childbirth experience ratings were observed (Fig. 1). NRS ratings 1–4 corresponded to a negative experience, NRS ratings 5–6 to a mixed experience, and NRS ratings 7–10 to a positive childbirth experience. In the empirical CDF graphs, the distribution of negative, mixed and positive overall childbirth experience was plotted against the scores on each of the four CEQ2 subscales.

Pearson correlation analyses were carried out to test the bivariate associations between CEQ2 domains and the NRS scores, and presented as Pearson correlation coefficients (*r*). To further explore the relationship between the four domains of the CEQ2 and overall childbirth experience, a multiple linear regression analysis was conducted, and presented as unstandardized beta coefficients (*B*) and 95% confidence intervals (CI). All assumptions for multiple linear regression were fulfilled. Statistical analyses were performed using IBM SPSS Statistics version 28.0 (IBM Corp., Armonk, N.Y., USA).

## Results

### Sample characteristics

A total of 2953 women completed the CEQ2 questionnaire; 2820 from the university referral hospital, (corresponding to 62% of women with spontaneous or induced onset of labour who rated overall childbirth experience during the recruitment period) and 133 from the regional hospital (corresponding to 17% of the eligible women). Overall, the respondents’ mean age was 31.1 years (SD 4.4), and the majority were born in Sweden (74.6%). Around half of the women (51.3%) were parous, and among them, six percent (6.0%) had previously given birth by CS. Mean gestational week at birth was 39.4 (SD 1.7) with a range of 22 to 42 weeks. There were some small differences noted in the sample characteristics between the two study sites. Participants included at the regional hospital had a somewhat lower educational level, higher body mass index and more often reported poor self-rated health, compared to women from the university referral hospital. However, due to the small sample at this site, the sample characteristics are shown for the total sample in Table 1.

**Table 1:** Background-, pregnancy- and labour characteristics of the study population

**Characteristics, *n* = 2953**	*mean [SD] or n (%)*
Age, years	31.1 [4.4]
≤ 19	12 (0.4)
20 − 34	2258 (76.5)
≥ 35	647 (21.9)
Missing	36 (1.2)
Swedish-born	
No	453 (15.3)
Yes	2202 (74.6)
Missing	298 (10.1)
Country of birth by income level^a^	
High	2367 (80.2)
Upper middle	142 (4.8)
Lower middle	55 (1.9)
Low	91 (3.1)
Missing	298 (10.1)
Education, years	
> 12	1765 (59.8)
10–12	1040 (35.2)
≤ 9	98 (3.3)
Missing	50 (1.7)
Civil status	
Cohabitant	2770 (93.8)
Single mother	46 (1.6)
Other situation^b^	55 (1.9)
Missing	82 (2.8)
BMI at first antenatal visit, kg/m^2^	25.5 [5.0]
< 18.5	45 (1.5)
18.5 − 24	1374 (46.5)
25 − 29	736 (24.9)
≥ 30	423 (14.3)
Missing	375 (12.7)
Pre-pregnancy self-rated health	
Good	2308 (78.2)
Poor	245 (8.3)
Missing	400 (13.5)
Parity	
Primiparous	1421 (48.1)
Parous	1514 (51.3)
Missing	18 (0.6)
Previous CS^c^	
No	2530 (85.7)
Yes	176 (6.0)
Missing	247 (8.4)
Counselling for fear of childbirth	
No	2590 (87.7)
Yes	313 (10.6)
Missing	50 (1.7)
Induction of labour	
No	1937 (65.6)
Yes	893 (30.2)
Missing	123 (4.2)
Gestational week at birth	39.4 [1.7]
Missing	30 (1.0)
Multifetal birth (twins)	
No	2845 (96.3)
Yes	31 (1.0)
Missing	77 (2.6)
Mode of birth	
Spontaneous vaginal	2467 (83.5)
Vacuum extraction	193 (6.5)
Unplanned CS	260 (8.8)
Immediate CS	19 (0.6)
Missing	14 (0.5)

*SD* Standard Deviation, *BMI* Body Mass Index, *CS* Caesarean Section

^a^Based on the 2018 World Bank country classifications

^b^In a relationship but living apart, or living with parents or extended family

^c^Of parous

### Overall childbirth experience

Mean overall childbirth experience among the women was 8.1 (SD 1.9). The distribution of values is illustrated in Fig. 1. The prevalence of negative childbirth experience was 6.3% in total sample, and 9.1% among primiparous women. Altogether, 8.8% of women reported a mixed overall childbirth experience, whereas the vast majority, 84.9%, reported a positive childbirth experience.

### Childbirth Experience Questionnaire 2

Descriptive statistics for CEQ2 subscales and total scale are shown in Table 2. Cronbach’s α-coefficients indicated high levels of internal consistency for the subscales *own capacity* and *perceived safety*, as well for the total CEQ2 scale, with α-values well above 0.70 (α = 0.78, α = 0.81, and α = 0.89, respectively). The subscales *professional support* and *participation* reached acceptable reliability, with α-values just below 0.70 (α = 0.69, and α = 0.66, respectively).

**Table 2:** Descriptive statistics for Childbirth Experience Questionnaire 2, shown as subscale and total scale scores, *n* = 2953

Subscales and items	Excluded in calculation of subscale score	Mean (SD)	Min	Max	Cronbach’s alpha	Excluded in calculation of Cronbach’s alpha
Own capacity	5	2.70 (0.56)	1.00	4.00	0.78	101
Labour and birth went as I had expected I felt strong during labour and birth I felt capable during labour and birth I was tired during labour and birth.^a^ I felt happy during labour and birth I felt that I handled the situation well As a whole, how painful did you feel childbirth was?^a^ As a whole, how much control did you feel you had during childbirth?						
**Perceived safety**	15	3.28 (0.62)	1.00	4.00	0.81	116
I felt scared during labour and birth.^a^ My impression of the team’s medical skills made me feel secure I have many positive memories from childbirth I have many negative memories from childbirth.^a^ Some of my memories from childbirth make me feel depressed.^a^ As a whole, how secure did you feel during childbirth?						
**Professional support**	23	3.71 (0.41)	1.20	4.00	0.69	76
Both my partner and I were treated with warmth and respect I would have preferred the midwife to be more present during labour and birth.^a^ I would have preferred more encouragement from the midwife.^a^ The midwife conveyed an atmosphere of calm The midwife helped me to find my inner strength						
**Participation**	8	3.70 (0.51)	1.00	4.00	0.66	41
I wish the staff had listened to me more during labour and birth.^a^ I took part in decisions regarding my care and treatment as much as I wanted I received the information I needed during labour and birth						
**Total scale**	30	3.35 (0.42)	1.27	4.00	0.89	221

^a^Item reversed in scoring

### Overall childbirth experience versus CEQ2

**Fig. 2:**
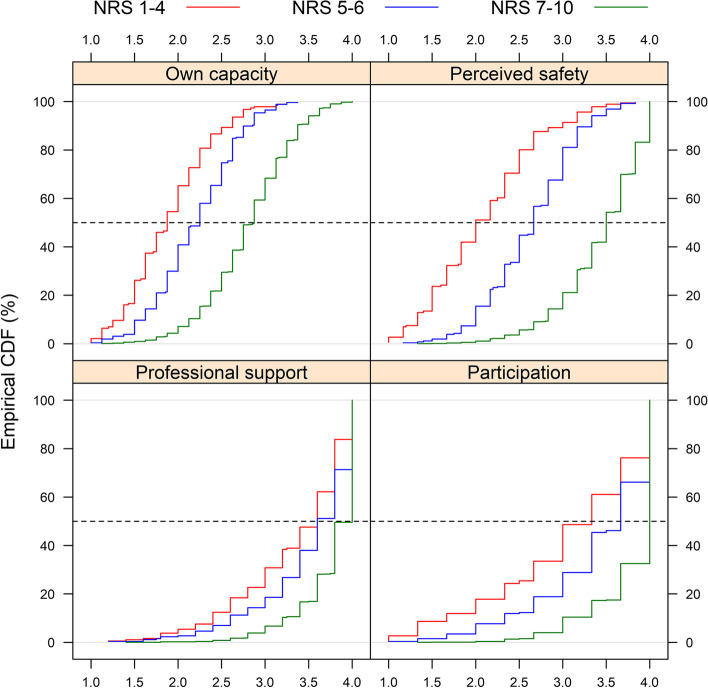
Empirical cumulative distribution function (CDF) graphs showing the distribution of overall childbirth experience stratified as negative (red, *n* = 187), mixed (blue, *n* = 260) and positive (green, *n* = 2506) on CEQ2 subscales Own capacity, Perceived safety, Professional support and Participation. The x-axes indicate scores on CEQ2 subscales ranging from 1 to 4, and the y-axes show the proportion of women with a certain score. Higher scores indicate better experiences within the respective CEQ2 subscale. The dotted lines indicate median values

The distribution of overall childbirth experience, stratified as negative, mixed and positive on CEQ2 subscales, is illustrated in Fig. 2. Women displayed greater group-wise variety in their experiences regarding *own capacity* and *perceived safety*, compared to experiences representing *professional support* and *participation,* as shown by the greater distance between the lines representing negative, mixed and positive overall childbirth experience in the upper two graphs (Fig. 2)*.* Regardless of overall childbirth experience, the majority of respondents scored high on the CEQ2 subscales *professional support* and *participation.*

Pearson correlation analyses showed moderate to strong correlations between NRS and CEQ2 (total scale: *r* = 0.69, *own capacity: r* = 0.58, *perceived safety: r* = 0.71, *professional support: r* = 0.38 and *participation: r* = 0.46, all at *p* < 0.05). Multiple linear regression analyses shown in Table 3 showed that overall childbirth experience in this sample was mainly explained by *perceived safety* (*B* = 1.60, CI 1.48–1.73), followed by *own capacity* (*B* = 0.65, CI 0.53–0.77) and *participation* (*B* = 0.43, CI 0.29–0.56). For every scale score increase on the *perceived safety* subscale, women scored 1.6 scores higher on the NRS measuring overall childbirth experience, while one more scale score increase on the *participation* subscale only raised NRS ratings by 0.43. Adjusting for ratings on all other subscales, the subscale *professional support* did not explain ratings of overall childbirth experience (*B* = 0.05, CI -0.10–0.21). The model including the four subscales of the CEQ2 explained 52.3% of the variance in NRS ratings of overall childbirth experience.

**Table 3:** Linear multiple regression analysis between CEQ2 subscales and overall childbirth experience (1–10), *n* = 2923

Subscale	*B* coefficient (95% CI)	*P*-value
Own capacity	0.65 (0.53–0.77)	< 0.001
Perceived safety	1.60 (1.48–1.73)	< 0.001
Professional support	0.05 (-0.10–0.21)	0.506
Participation	0.43 (0.29–0.56)	< 0.001
*R*^*2*^	0.523	< 0.001

*CI* confidence interval

## Discussion

In this study, ratings of overall childbirth experience with a single-item measurement were examined based on the dimensions included in the CEQ2 among close to 3000 women. Overall childbirth experience ratings were mainly explained by the dimension *perceived safety*, and to a lesser extent by *own capacity* and *participation*. It was not explained by the dimension *professional support*. This is one of the first studies to explore overall childbirth experience in three categories, including a definition of mixed experience. Viewing childbirth experience more on a continuum, and not only as the customary negative and positive experience, has recently been encouraged [27].

Irrespective of overall childbirth experience, women reported high levels of *professional support*. This could partly be an effect of social desirability bias, since women were asked about interactions with their care providers while still at the hospital, but could also be seen in the light of CEQ2 having persistent ceiling effects, also after revision of the first version of the Childbirth Experience Questionnaire (CEQ) [23]. Regardless of the reasons for this ceiling effect, the NRS rating of childbirth experience appeared not to reflect *professional support* as measured by CEQ2. In a context where the majority of women rated professional support high, NRS mainly appeared to capture the domain *perceived safety.* The importance of feeling safe and secure during labour is well known in relation to birth experience, and it has been suggested that the relation to the midwife is a key to this [28]. In the present study, the midwife’s presence in itself, an example of professional support recently described by the term “watchful attendance” [29], may have created a sense of calm and security, and thereby raised the woman’s perceived safety. Since support is by its nature something provided to the woman, rather than an experience in itself, professional support may also have been indirectly captured in the NRS values by the other CEQ2 subscales. Future studies on how to best measure professional support are needed.

Comparisons of NRS and CEQ2 at later time points could possibly show different results, since the perception of childbirth experience may change over time [30]. In a small, longitudinal study (*n* = 78), satisfaction with childbirth at one week and three months postpartum, using VAS ratings, converted to a 10-point NRS, and CEQ were compared [24]. In line with the present results, the NRS rating was independently associated with the domains *perceived safety* and *own capacity* at one week postpartum, but not with *professional support* or *participation*. Moreover, the present results are partly in line with findings from a recent study, where VAS ratings, converted to an 11-point NRS, and CEQ were compared regarding negative childbirth experience in a sample of women all having had their labour induced (*n* = 362) [25]. VAS was completed on one of the first days after childbirth, and CEQ up to a month postpartum. In this study, negative childbirth experience, defined as NRS ratings 0–4, mainly reflected low levels of *perceived safety* and *own capacity,* to a lesser extent *professional support*, but not* participation.* Similar findings in these studies were observed despite differences in study design, indicating that rating of overall childbirth experience indeed captures mainly *perceived safety* and *own capacity,* rather than *professional support* and* participation.*

Regarding the internal consistency for CEQ2 in this setting, Cronbach’s α was almost equivalent to the values from the validation of the original CEQ2 for the total scale (α = 0.89 compared to α = 0.91) and subscales *own capacity* and *perceived safety* (0.78 and 0.81 compared to 0.82 and 0.83), whereas Cronbach’s α for subscales *professional support* and *participation* differed more from the original CEQ2 (0.69 and 0.66 compared to 0.76 and 0.73). The CEQ2 was originally validated for use at home, three to four weeks postpartum, by women with spontaneous onset of labour. In the present study, the subscales *professional support* and *participation* had slightly lower reliability compared to the validation study [23]. The somewhat lower alpha scores for those subscales could indicate lower inter-relatedness between the individual questions in those domains in this sample. However, reliability was still acceptable, justifying the use of CEQ2 also shortly after childbirth and including both women with spontaneous onset and whose labour was induced. In a sensitivity analysis including only inductions results remained largely the same, though with some minor improvement of Cronbach’s alphas for the subscales *professional support* and *participation* (Supplementary table 1).

The prevalence of negative childbirth experience, 6.3% in total sample and 9.1% among primiparas, is consistent with previous studies in comparable settings [2, 3, 5]. Prevalence of negative childbirth experience according to CEQ2 was not calculated, since no cut-off exists for the instrument. Hence, no comparisons in prevalence of negative childbirth experience depending on use of instrument were possible. Previous research, on experiences of intrapartum care, suggests that more extensive instruments give a more negative picture of childbirth experiences, compared to single-item measurements [31]. Indications of this were proposed in a study using CEQ2 and a definition of negative childbirth experience as values > 1 SD below mean score of the population, resulting in a prevalence of negative childbirth experience of 37% [32]. To establish a cut-off for negative childbirth experience according to CEQ2 would be desirable, especially since the developers of CEQ2 suggest use of the instrument as a tool for identifying women with negative childbirth experiences [22]. Future studies could explore the use of CEQ2 in clinical settings, and aim to establish clinically useful cut-offs for both NRS and CEQ2.

### Strengths and limitations

In this study, NRS was examined against a well-established and recommended childbirth experience-measuring instrument, and the full range of childbirth experiences were explored, not only negative ones, as in previous studies [25]. The CEQ2 was completed at the same time as the rating of overall childbirth experience, which minimises the risk of recall bias. The ratings of overall childbirth experience and completion of the CEQ2 were carried out at the birth clinic, which could have influenced participants’ responses by introducing social desirability bias. However, it is important to note that data were collected by midwifes and assistant nurses not involved during labour and childbirth.

One major strength of this study is the sample size, covering a wide range of NRS ratings of overall childbirth experience from hospitals of different sizes and locations (urban and rural). To facilitate data collection for recruiting staff at the study sites, no information was collected on reasons for non-participation. An exact inclusion rate was therefore not possible to determine. Also, no records were kept on how many women were unable to participate due to a language barrier. Some women may have had sufficient language skills for rating overall childbirth experience on the NRS, but were not fluent enough in Swedish to answer the more extensive CEQ2. The CEQ and/or the CEQ2 are available in several languages, but no translated versions of the questionnaire were used in the present study besides the Swedish version [33–35]. One reason for this was that available language versions did not correspond to the languages most common among migrants in Sweden at the time of data collection. Also, the translated versions were not validated for use in a Swedish setting, which could have complicated interpretation of the results. Thus, the exclusion of non-Swedish-speaking women is a limitation of the study, which has to be considered when interpreting the results.

Although a high response rate was achieved at one of the study sites (62% at the university referral hospital), fewer women (17%) participated at the regional hospital. This is likely due to the main responsible data collector’s proximity to the university referral hospital, and the already existing routine there to distribute paper questionnaires, since NRS ratings were collected on paper also prior to the study period. At the regional hospital, NRS ratings were collected verbally, and the distribution of paper questionnaires an additional procedure during the study period. Moreover, the shorter period of data collection at the regional hospital, due to a delayed study start related to COVID-19 pandemic restrictions, may have affected the inclusion rate by allowing a shorter time period of data collection after the usually slower starting-up period. However, only small differences in sample characteristics were detected between study sites, and the total sample is comparable to the childbearing population in Sweden which strengthens generalisability [36].

## Conclusions

In conclusion, overall childbirth experience rated by a single-item global measurement appears to mainly capture experiences of *perceived safety* and to a lesser extent *own capacity* and *participation.* On the other hand, it appears not to reflect *professional support*. CEQ2 shows good psychometric properties for use shortly after childbirth among women with spontaneous and induced onset of labour, which increases the usability of the instrument. NRS is an easy and convenient method for collecting childbirth experiences, but mainly regarding *perceived safety* and *own capacity.* Thus, one needs to be aware that NRS does not capture the whole experience since, although these are valuable dimensions of the childbirth experience, they are not comprehensive. These results suggest that future efforts to improve care in connection to childbirth may advantageously focus on interventions to strengthen women’s sense of safety and capability, in order to prevent consequences of negative childbirth experience, but further studies are warranted to address this issue.

## Supplementary Information

**Additional file 1: Supplementary table 1.** Descriptive statistics for Childbirth Experience Questionnaire 2, shown as subscale and total scale scores, stratified on spontaneous or induced onset of labour, *n* = 2830.

## Data Availability

The data that support the findings of this study are available from the corresponding author upon reasonable request.

## Abbreviations

α: Alpha
*B*: Beta coefficient
CEQ: Childbirth Experience Questionnaire
CEQ2: Childbirth Experience Questionnaire 2
CS: Caesarean Section
CDF: Cumulative Distribution Function
NRS: Numeric Rating Scale
SD: Standard Deviation
VAS: Visual Analogue Scale
