# Supplementary material for: Overall childbirth experience: what does it mean? A comparison between an overall childbirth experience rating and the Childbirth Experience Questionnaire 2
Source: BMC Pregnancy Childbirth. 2023 Mar 14;23:176. doi: 10.1186/s12884-023-05498-5 (PMC10012290; doi:10.1186/s12884-023-05498-5)
Supplement: Supplementary file 1 — Additional file 1: Supplementary table 1. Descriptive statistics for Childbirth Experience Questionnaire 2, shown as subscale and total scale scores, stratified on spontaneous or induced onset of labour, n = 2830. [file 12884_2023_5498_MOESM1_ESM.docx]

**Supplementary table 1.** Descriptive statistics for Childbirth Experience Questionnaire 2, shown as subscale and total scale scores, stratified on spontaneous or induced onset of labour, n = 2830.

| **Induced onset of labour,**  **n = 893** | | | | | | |
| --- | --- | --- | --- | --- | --- | --- |
| Subscale | Excluded in calculation of subscale score | Mean (SD) | Min | Max | Cronbach’s alpha | Excluded in calculation of Cronbach’s alpha |
| Own capacity | 3 | 2.65 (0.56) | 1.00 | 4.00 | 0.79 | 35 |
| Perceived safety | 7 | 3.22 (0.65) | 1.00 | 4.00 | 0.82 | 38 |
| Professional support | 8 | 3.68 (0.45) | 1.20 | 4.00 | 0.72 | 30 |
| Participation | 2 | 3.67 (0.56) | 1.00 | 4.00 | 0.70 | 13 |
| Total scale | 13 | 3.31 (0.45) | 1.27 | 4.00 | 0.90 | 77 |
| **Spontaneous onset of labour,**  **n = 1937** | | | | | | |
| Subscale | Excluded in calculation of subscale score | Mean (SD) | Min | Max | Cronbach’s alpha | Excluded in calculation of Cronbach’s alpha |
| Own capacity | 2 | 2.73 (0.55) | 1.00 | 4.00 | 0.78 | 58 |
| Perceived safety | 7 | 3.31 (0.60) | 1.00 | 4.00 | 0.81 | 70 |
| Professional support | 14 | 3.73 (0.39) | 1.20 | 4.00 | 0.67 | 42 |
| Participation | 6 | 3.72 (0.48) | 1.00 | 4.00 | 0.65 | 27 |
| Total scale | 16 | 3.37 (0.40) | 1.59 | 4.00 | 0.88 | 130 |

SD = Standard Deviation.
